# Assessing the *Cntnap2* knockout rat prepulse inhibition deficit through prepulse scaling of the baseline startle response curve

**DOI:** 10.1038/s41398-023-02629-6

**Published:** 2023-10-18

**Authors:** Alaa El-Cheikh Mohamad, Dorit Möhrle, Faraj L. Haddad, Anton Rose, Brian L. Allman, Susanne Schmid

**Affiliations:** 1 Anatomy & Cell Biology, Schulich School of Medicine & Dentistry, University of Western Ontario, London, ON Canada; 2 Department of Psychology, University of Western Ontario, London, ON Canada

**Keywords:** Learning and memory, ADHD

## Abstract

Many neurodevelopmental disorders, including autism spectrum disorder (ASD), are associated with changes in sensory processing and sensorimotor gating. The acoustic startle response and prepulse inhibition (PPI) of startle are widely used translational measures for assessing sensory processing and sensorimotor gating, respectively. The *Cntnap2* knockout (KO) rat has proven to be a valid model for ASD, displaying core symptoms, including sensory processing perturbations. Here, we used a novel method to assess startle and PPI in *Cntnap2* KO rats that allows for the identification of separate scaling components: startle scaling, which is a change in startle amplitude to a given sound, and sound scaling, which reflects a change in sound processing. *Cntnap2* KO rats show increased startle due to both an increased overall response (startle scaling) and a left shift of the sound/response curve (sound scaling). In the presence of a prepulse, wildtype rats show a reduction of startle due to both startle scaling and sound scaling, whereas *Cntnap2* KO rats show normal startle scaling, but disrupted sound scaling, resulting in the reported PPI deficit. These results validate that startle and sound scaling by a prepulse are indeed two independent processes, with only the latter being impaired in *Cntnap2* KO rats. As startle scaling is likely related to motor output and sound scaling to sound processing, this novel approach reveals additional information on the possible cause of PPI disruptions in preclinical models.

## Introduction

Sensory processing and sensory filtering are basic building blocks for cognitive function. Alterations in sensory processing and filtering are common in individuals with neurodevelopmental disorders, including autism spectrum disorder [1–3]. A common paradigm used for assessing sensory filtering and sensorimotor gating in both human and animal models is the acoustic startle response (for review, see ref. [4]) and the attenuation of startle by a prepulse (Prepulse Inhibition or PPI, for review see [5]). The neural pathways involved in startle and PPI have been investigated through diverse studies utilizing lesioning [6, 7], decortication [8], behavioral investigations [9–12], optogenetics [13–16], chemogenetics [17], pharmacological investigations [18–22], as well as electrical stimulation [23]. While the startle pathway has been well defined as a highly conserved short pathway in the brainstem (for review see [4, 24]), PPI is thought to be mediated through a feed-forward mechanism originating in the brainstem and involving midbrain and higher-order brain structures (Supplemental Fig. 1; for review see [5]).

**Fig. 1:**
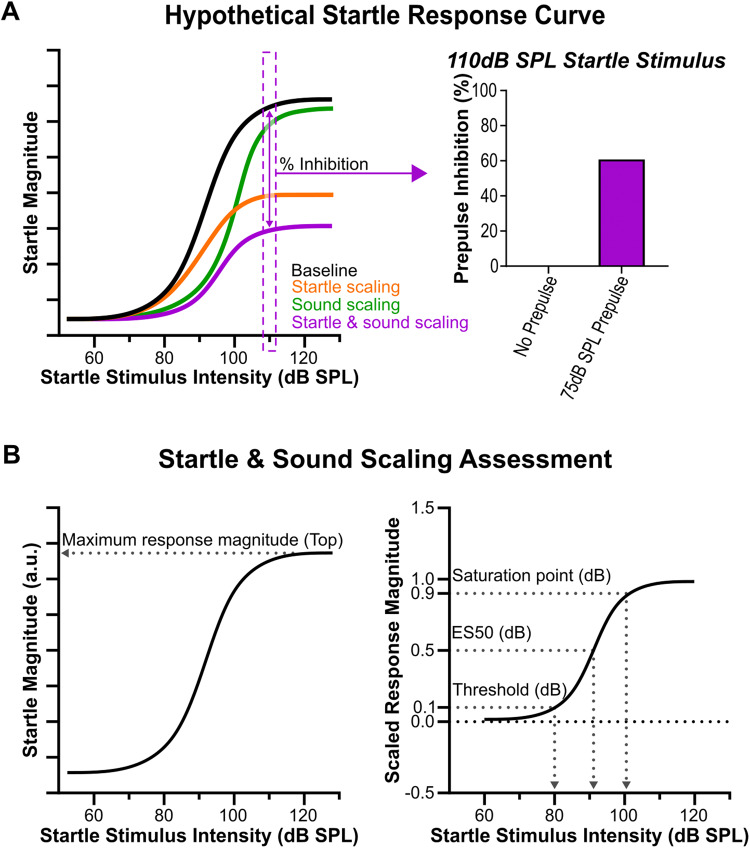
Hypothetical baseline startle response curve. **A** Baseline startle response curve (black) illustrating how a prepulse hypothetically scales the curve. Startle scaling (orange) results in a downward shift and sound scaling (green) a rightward shift of the baseline startle curve. Taken together startle- and sound scaling result in the scaled response curve (purple) which classical analyses (as seen on the right) cannot show with a single startle stimulus intensity [12]. **B** Hypothetical baseline startle response curve visualizing parameters extracted from the sigmoidal regression used in the scaling analysis. The maximum response (Top) was used to assess startle scaling (left). The threshold, ES50, and saturation points were used to assess sound scaling from a normalized curve (right).

Although PPI is a very commonly used metric in behavioral neuroscience, reported PPI deficits have been notoriously inconsistent in many animal models and patient populations [10, 25]. Miller and colleagues [12] have recently proposed an improved protocol for assessing startle and PPI [12], suggesting that the entire baseline startle response curve is scaled by the prepulse [12]. The baseline startle response curve is a sigmoidal input/output (I/O) function, relating startle stimulus intensity (input) to the corresponding response magnitude (output; Fig. 1A). Various parameters, such as the reflex capacity, efficiency, and threshold, can be extracted from the sigmoidal function which reflect sensory processing and motor output mechanisms [11, 12]. Miller et al. [12] defined changes in the startle I/O curve due to a prepulse as different scaling components, which shift the curve in both a downward and a rightward direction. These components are referred to as startle scaling, a reduction in response amplitude, reflected by a change in the reflex capacity ([11, 12] Fig. 1B), and sound scaling, a reduction in sound sensitivity, reflected by changes in both stimulus potency and response threshold [11]. Startle scaling is related to motor output, and likely occurs in the primary startle pathway, more specifically at the level of the caudal pontine reticular nucleus (PnC; see Supplemental Fig. 1, [7, 26]), whereas sound scaling involves a change in sound processing, and likely occurs in the PPI pathway in structures upstream of PnC. Taken together these two scaling components and their parameters are thought to describe the full effect of a prepulse on the baseline startle response curve [11, 12], which classical methods using a single startle stimulus intensity cannot convey ([10], Fig. 1A).

In this study we used both classical PPI analysis [27] and a variation of the protocol suggested by Miller et al. [12] to assess the scaling of the baseline startle response curve in the *Cntnap2* homozygous knockout (KO) rat, a well-established model for neurodevelopmental disorder, which has been consistently reported to show hyperreactivity to sound and a deficit in PPI [28–30]. We hypothesize that scaling is differentially affected in *Cntnap2* KO rats, which would not only validate a new approach to measure PPI, but also indicate where the underlying neural changes causing altered startle are likely occurring.

## Materials and methods

### Animals

Adult (3.5–6.5 months old) male (M) and female (F) Sprague Dawley wildtype (*Cntnap2*^*+/+*^; M = 15, F = 13) and homozygous KO (*Cntnap2*^*−/−*^; M = 14, F = 12) littermates were used. Rats from 16 litters were obtained through in-house breeding of heterozygous crossings (*Cntnap2*^*+/−*^ × *Cntnap2*^*+/−*^). Rats were housed in groups of 2–3 animals per cage, while some animals known to be seizure-prone were housed alone to minimize stress to the rat and cage-mates. Seizure-prone animals were included in testing and data analyses as seizures are a known phenotype of *Cntnap2* loss-of-function mutation in the rodent model [31] as well as in humans [32]. Rats were housed in open-top cages insulated with bedding made of wood shavings and enriched with wrinkle paper and plastic huts. Additionally, rats had access to food and water *ad libitum* and were kept on a 12-hour light/dark cycle with testing occurring during the light phase (7:00 to 19:00 hours). Experimental procedures were approved by the University of Western Ontario Animal Care Committee and were in accordance with guidelines set by the Canadian Council on Animal Care.

### Behavioral testing—startle responses

Acoustic startle responses were measured using the Med Associates (Vermont, USA) startle system; in the startle chambers, rats were placed in perforated, non-restrictive plexiglass tubes on weight-transducing platforms. Prior to behavioral testing, rats were handled and acclimated to the testing environment for 5 minutes with only background noise (65 dB sound pressure level, SPL, white noise), followed by ~3 minutes of handling by the experimenter. This acclimation and handling procedure was repeated three times to build familiarity with the experimenter and environment. On the final day of acclimation, an I/O function was performed to determine the startle reactivity of each animal. By determining startle reactivity, adjustments to the gain setting of the platform for each animal could be made allowing for an optimal reading of the startle waveform and avoiding the loss of responses that may occur above or below the software’s recording limits. The I/O function involved the presentation of startle stimuli ranging from 65 dB to 120 dB (20 ms white noise) increasing in increments of 5 dB, in addition to the prepulse stimuli used in the PPI protocol (75 dB, 80 dB, and 85 dB, 4 ms white noise); stimuli were presented in a pseudorandomized order. Cages were changed following I/O and were not changed until after the final PPI session to ensure that cage change stress was not a factor in the reactivity of animals during testing.

### Behavioral testing—modified PPI protocol

Modifications to a classic PPI testing paradigm were made in accordance with Miller et al. [12]. Traditionally, a PPI protocol involves the use of two prepulse stimuli, a single startle stimulus, and two interstimulus intervals (ISI); this paradigm is conducted twice with each prepulse-startle stimulus-ISI combination presented ten times per testing session (for more see [27]). The novel advanced PPI paradigm involved three consecutive procedural blocks. In first block, animals were acclimated to the startle chamber for 5 mins with only background noise (65 dB white noise). The following habituation block involved the presentation of a 110 dB (20 ms white noise) startle stimulus over 12 trials with a variable intertrial interval of 10-15 seconds. In the final PPI block, a non-startling prepulse (75 dB, 80 dB, or 85 dB, 4 ms white noise) was paired with one of five startle stimuli (80 dB, 90 dB, 100 dB, 110 dB, or 120 dB, 20 ms white noise) at a fixed ISI of 100 ms; a startle-alone condition was also presented. Trials were separated by a variable intertrial interval of 10-15 seconds and presented in a pseudorandomized order. Each condition was repeated 4 times per testing session, resulting in 80 trials per session. This procedure was repeated twice a day—with daily sessions separated by a minimum of 4 hours—over 5 days for a total of 10 testing sessions and 40 trials for each condition.

### Data analysis

Startle response values were adjusted for each rat by the gain factor prior to statistical analyses, allowing for startle magnitude comparisons between animals. The startle magnitude was determined to be the maximum peak-to-peak value (highest crest and lowest trough) of the response waveform generated by the body flinch following the presentation of the startling stimulus. Percent PPI was calculated from startle magnitudes obtained in the PPI block:

$$\% {\rm{PPI}}=\left(1-\frac{{\rm{startle}}\,{\rm{magnitude}}\,{\rm{with}}\,{\rm{prepulse}}}{{\rm{baseline}}\,{\rm{startle}}\,{\rm{magnitude}}}\right)\times 100 \%$$

For the advanced PPI method, statistical analysis of changes to the baseline startle response curve due to prepulses was conducted in accordance with Martin–Iverson and Stevenson [11] and the scaling components defined by Miller et al. [12]. Startle reactivity was assessed over the range of startle stimuli by fitting each animal’s responses to a sigmoidal regression function in GraphPad Prism 9.3.1 (San Diego, California, USA; Non-linear regression; Method: Sigmoidal, 4PL, X is concentration; Method: Least squares regression; Initial values: choose automatically; Confidence: Unstable parameters and ambiguous fits as Neither option; Diagnostics: default values including Adjusted R Squared, RMSE, and tests of normality; see also [28]). The sigmoidal regression formula produced the parameters of interest for evaluating changes in the curve due to a prepulse:

$$Y={Bottom}+{X}^{{Hillslope}}\left(\frac{{Top}-{Bottom}}{{X}^{{Hillslope}}+{{ES}50}^{{Hillslope}}}\right)$$

where Y is the startle response magnitude, Bottom is the minimum startle response magnitude, and Top is the maximum startle response magnitude; these parameters correspond with the y-axis of the curve. X is the startle stimulus intensity (dB SPL) required to produce a certain Y value (in arbitrary units), and ES50 is the sound intensity (dB SPL) required to maintain the half-maximum response. Hillslope is the slope of the curve.

To assess startle scaling, the Bottom parameter was forced to zero by subtracting movement to background noise before prepulse onset from the startle response magnitude based on the prepulse curve to be fit. Each animal’s responses were then fit to a sigmoidal regression function as described above (including, Constrain: Bottom is constant equal to 0). Startle scaling was thus determined to be a change in the maximum startle response (Top).

To evaluate sound scaling, startle responses for each animal and prepulse condition were scaled between 0 (movement during background noise) and 1, these scaled values were then fit to the sigmoidal regression function using the same procedure as above (except, Constrain: Bottom is constant equal to 0 and Top is constant equal to 1). Sound scaling was determined to be a change in the threshold [11, 28], ES50 [11], and saturation point [11, 28]; ES50 was provided by the regression. Threshold and saturation point were calculated in MATLAB R2022a by re-arranging Equation 2 to solve for X. The threshold Y value was set to 10% of the Top, and saturation point Y value was set to 90% of the Top.

$$X=\root{Slope}\of{\frac{(Y-{Bottom})\times ({{ES}50}^{{Slope}})}{{Top}-Y}}$$

### Statistical analysis

Both classical and elaborate data analyses were conducted in GraphPad Prism 9.3.1. Classical analyses included comparing baseline acoustic startle responses at each startle stimulus intensity and %PPI between genotypes. Elaborate analyses involved fitting data with a non-linear sigmoidal regression to assess the extracted parameters. Data across sexes was pooled into genotype as classical analyses revealed no differences between sexes within each genotype (see Supplemental Figs. 3 and 4 for sex-specific data). The same animals were included in both analysis methods.

Outlier analysis was conducted in IBM SPSS (version 26) with the raw startle response magnitudes for each condition and genotype. Extreme outliers, >3 interquartile ranges (IQR) from the median, were identified through boxplot assessment and excluded from all analyses; four *Cntnap2* WTs (M = 1, F = 3) and four *Cntnap2* KOs (M = 3, F = 1) were found to be extreme outliers and removed. The Shapiro-Wilk test of normality was used to determine the shape of startle response distributions to specific sounds at the individual animal level. Due to the lack of normal distributions within individual animal responses, median values were used in the statistical analyses and calculation of %PPI. Consequently, non-parametric statistics were performed, as lack of normality violates the assumptions of parametric tests and medians cannot be used in these types of tests [33, 34]. Figures are presented as median values with error bars indicating IQR. The following statistical tests were used: Shapiro-Wilk test, Kruskal-Wallis test, Friedman test, Mann-Whitney *U* test, and Spearman’s correlation. Statistical tests used in the classical analyses were corrected for multiple comparisons using the Holm-Sidak method. Tests were followed by post hoc Dunn’s test of multiple comparisons when appropriate. Alpha was set to statistical significance *α* = 0.05; *p* values, which can be found in figure captions, are represented as: no asterisk for non-significance, *p < 0.05, **p < 0.01, ***p < 0.001, ****p < 0.0001.

## Results

### Normality of startle response amplitudes is dependent on testing conditions

Classical startle analyses use the average startle response of an individual animal, usually calculated from ten or more responses, although response amplitudes are often not normally distributed. Miller et al. [12] suggest that a log_10_ transformation is necessary to improve data normality. In order to test if a log_10_ transformation indeed improves the normality of our startle data, we assessed startle response distributions for normality using the Shapiro–Wilk test for each trial type (baseline startle, prepulse-startle) within individual animals. We quantified the proportion of the experimental groups with normally distributed startle responses for each trial type (*n* = 46) using the raw startle magnitude data and the log_10_ transformed startle magnitudes (see Supplementary Table 1).

The proportion of the population with normally distributed startle responses using the raw data ranged from 0% to 52.17% depending on the condition, while the log_10_ transformed startle magnitude proportions ranged from 8.70% to 80.43% (Supplementary Table 1). Although, it may seem more favorable to use the log_10_ transformed data due to the higher level of normality, it should be noted that the log_10_ transformation actually reduced normality for several conditions, including for the baseline startle responses at relevant startle stimulus intensities (100 dB, 110 dB, and 120 dB). In other words, a greater proportion of the population showed normally distributed baseline startle responses when using the raw data, rather than the log_10_ transformation (Supplementary Table 1 and Supplementary Fig. 2). Since all %PPI is calculated using these baseline startle responses, the use of log_10_ transformed data may skew statistical analyses of PPI. We therefore decided to use a conservative approach and to not log_10_ transform startle data. However, since a large portion of the data is not normally distributed, we used the median, a non-parametric measure of central tendency that is unaffected by skew and outliers and does not assume normal distribution of the data [33, 34], rather than the classically used average, throughout our data analysis.

### *Cntnap2* KO rats show increased baseline startle responses and deficient PPI

To validate the use of medians instead of means, and to ensure consistency with previous findings of altered sensory processing in the *Cntnap2* KO rat [29, 30], baseline startle responses and PPI were first analyzed in the classical method.

**Fig. 2:**
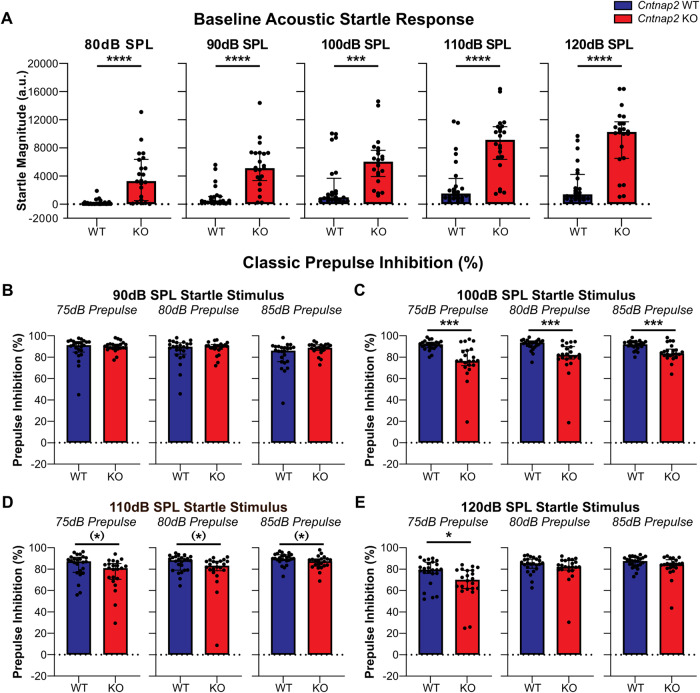
Classical assessment of startle and PPI. *Cntnap2* WTs are represented in blue and *Cntnap2* KOs in red. Scatter plots represent individual animals and bars represent group medians with error bars as IQR. **A** Baseline acoustic startle response magnitudes for startle-alone conditions (left to right: 80 dB, 90 dB, 100 dB, 110 dB, and 120 dB) compared between genotypes. *Cntnap2* KOs were hyperreactive to all startle stimuli in comparison to their WT counterparts. **B**–**E** Classical %PPI analysis at increasing startle stimulus intensities (90 dB, 100 dB, 110 dB, and 120 dB) with the three prepulse intensities (left to right: 75 dB, 80 dB, and 85 dB). **B** At the 90 dB startle stimulus, no differences were found between genotypes with all prepulse stimulus intensities. **C** At the 100 dB startle stimulus, *Cntnap2* KOs had a deficit in %PPI with the 75 dB, 80 dB, and 85 dB prepulse intensities in comparison to *Cntnap2* WTs. **D** At the 110 dB startle intensity, there was a statistical trend to significance with slightly reduced %PPI in *Cntnap2* KOs with all three prepulse stimuli. **E** At the 120 dB startle stimulus intensity, *Cntnap2* KOs had reduced %PPI with the 75 dB prepulse, however, no differences were found between the genotypes with 80 dB and 85 dB prepulse stimuli. Adjusted *p* value using Holm-Sidak correction for multiple comparisons; *p < 0.05, ***p < 0.001, ****p < 0.0001, no asterisk indicates non-significance of the comparison, and (*) trend towards significance.

Baseline startle responses were compared between genotypes at the five startle stimulus intensities employed (80 dB, 90 dB, 100 dB, 110 dB, and 120 dB) using the Mann–Whitney *U* test for WT vs KO with a Holm-Sidak correction for multiple comparisons. *Cntnap2* KOs showed hyperreactivity to sound at all startle stimulus intensities in comparison to WTs (80 dB: *p* < 0.0001; 90 dB: *p* < 0.0001; 100 dB: *p* = 0.0002; 110 dB: *p* < 0.0001; 120 dB: *p* < 0.0001; Fig. 2A and supplementary Table 2).

PPI was calculated and assessed with the classical %PPI equation where the median startle response in the presence of a prepulse is subtracted from the baseline startle response and PPI expressed as % inhibition from baseline startle. *Cntnap2* KOs showed a deficit in %PPI in comparison to WTs at higher startle stimulus intensities (Fig. 2B–E and Supplementary Table 3). More specifically, at the 90 dB startle stimulus intensity, no significant differences were found in %PPI between the genotypes with all three prepulse stimulus intensities (75 dB: *p* = 0.9465; 80 dB: *p* = 0.9465; 85 dB: *p* = 0.1123; Fig. 2B). At the 100 dB startle stimulus intensity, *Cntnap2* KO rats showed reduced %PPI in comparison to WTs with all three prepulse stimulus intensities (75 dB: *p* = 0.0002; 80 dB: *p* = 0.0002; 85 dB: *p* = 0.0009; Fig. 2C). At the 110 dB startle stimulus intensity, there was a statistical trend to significance with slightly reduced %PPI in *Cntnap2* KO rats with all prepulse conditions (75 dB: *p* = 0.0535; 80 dB: *p* = 0.0535; 85 dB: *p* = 0.0535; Fig. 2D). Finally, at the 120 dB startle stimulus intensity, *Cntnap2* KO rats displayed reduced %PPI with the 75 dB prepulse (*p* = 0.0301), but no significant difference was found between the genotypes with the higher prepulse stimulus intensities (80 dB: *p* = 0.2163; 85 dB: *p* = 0.1848; Fig. 2E). In summary, %PPI deficits were evident especially at startle stimulus intensities within the range typically used for startle and PPI testing and consistent with previous reports [5, 27].

### PPI is not dependent on baseline startle for either genotype

**Fig. 3:**
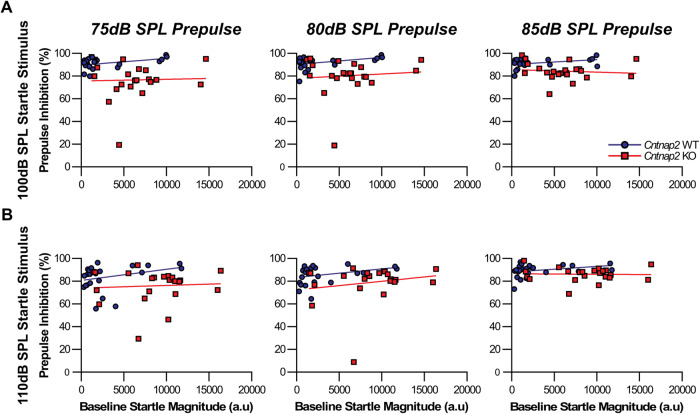
%PPI is not correlated with the baseline startle response for either *Cntnap2* WT or *Cntnap2* KO rats. Spearman’s correlations were conducted for the 100 dB and 110 dB startle stimuli with all three prepulse stimulus intensities (left to right: 75 dB, 80 dB, and 85 dB). *Cntnap2* WTs are represented in blue and *Cntnap2* KOs in red. Scatter plots depict individual values and lines of best fit were graphed using a simple linear regression. **A** 100 dB startle stimulus. No correlation was found between the baseline startle response and %PPI for either genotype with any of the prepulse stimulus intensities. **B** 110 dB startle stimulus. No correlation was found between the baseline startle response and %PPI for either genotype with any of the prepulse stimulus intensities.

As *Cntnap2* KOs were hyperreactive to the startle stimuli in comparison to WTs (Fig. 2A), we wanted to ensure this hyperreactivity was not a factor in the altered PPI. Therefore, Spearman’s correlations were conducted for the 100 dB and 110 dB startle stimuli for both WT and KO animals to assess if baseline startle and %PPI were correlated (Fig. 3). The line of best fit was graphed using a simple linear regression. No correlation was found for WT animals between baseline startle response and %PPI at the 100 dB (75 dB: *r* = 0.3652, *p* = 0.0793; 80 dB: *r* = 0.3965, *p* = 0.0551; 85 dB: *r* = 0.367, *p* = 0.0778; Fig. 3A) and 110 dB startle stimuli (75 dB: *r* = 0.3835, *p* = 0.0643; 80 dB: *r* = 0.3557, *p* = 0.0881; 85 dB: *r* = 0.3791, *p* = 0.0677; Fig. 3B) with any of the prepulse stimuli. Similarly, no correlation was found for *Cntnap2* KO rats between baseline startle response and %PPI for either the 100 dB (75 dB: *r* = −0.0774, *p* = 0.7322; 80 dB: *r* = −0.1056, *p* = 0.64; 85 dB: *r* = −0.2084, *p* = 0.3521; Fig. 3A) or 110 dB startle stimuli (75 dB: *r* = −0.0875, *p* = 0.6985; 80 dB: *r* = 0.0265, *p* = 0.9067; 85 dB: *r* = −0.0457, *p* = 0.8398; Fig. 3B) with any of the prepulse stimuli. Consistent with previous assessments of baseline startle and different measures of PPI [9], %PPI was independent of the baseline startle for either genotype, indicating that the higher baseline startle amplitude in *Cntnap2* KO rats is not related to the observed PPI deficit.

### *Cntnap2* KO baseline startle response curve is shifted up- and leftward

Using the advanced analysis as recently proposed (but with medians instead of log_10_ transformed data), baseline startle response curve parameters such as maximum response magnitude (Top), threshold, ES50, and saturation points, were extracted from sigmoidal regression fittings of the sound/response curves and assessed via Mann-Whitney *U* tests for WT vs KO animals. Startle response curves were first fit by restricting the bottom parameter of the curve to 0; from this fitting the maximum response magnitudes (Top) for each animal were extracted. Subsequently, startle response curves were normalized between 0 and 1, and sound levels for response threshold, ES50, and saturation points were extracted for each animal. Two animals (WT = 1; KO = 1) were excluded from the threshold, ES50, and saturation point comparisons because threshold and saturation points could not be calculated from their fitted curves.

**Fig. 4:**
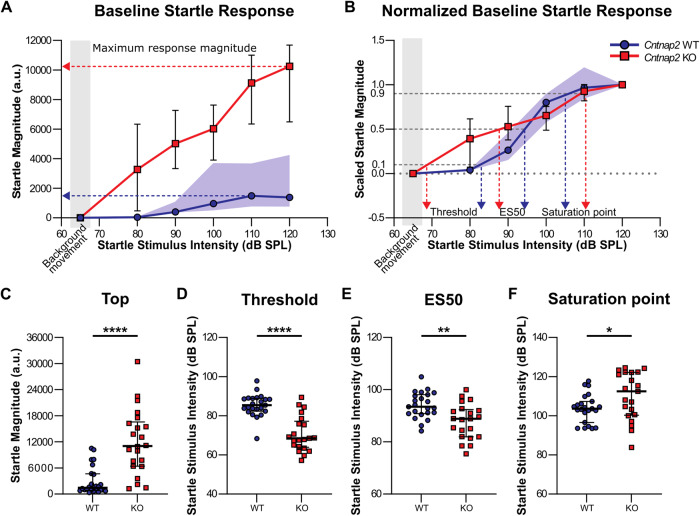
Comparison of *Cntnap2* WT and KO baseline startle response curves and parameters of interest. *Cntnap2* WTs are represented in blue and *Cntnap2* KOs in red. Scatter plots represent individual values and black lines represent median with error bars as IQR. **A** Startle response curve. Arrows point to the Top value for each group. *Cntnap2* WT IQR is visualized as the blue-shaded area. **B** Scaled startle response curve. Arrows point to the threshold, ES50, and saturation point values for each group. *Cntnap2* WT IQR is visualized as the blue-shaded area**. C** Top. *Cntnap2* KO rats have a greater maximum startle response magnitude than *Cntnap2* WTs. **D** Threshold. *Cntnap2* KO rats have a lower response threshold than *Cntnap2* WTs. **E** ES50. *Cntnap2* KO rats have a lower ES50 than *Cntnap2* WTs. **F** Saturation point. *Cntnap2* KO rats have a higher saturation point than *Cntnap2* WTs. *p < 0.05, **p < 0.01, ****p < 0.0001, no asterisk indicates non-significance of the comparison.

Congruent with classical analyses (Fig. 2A), KO animals displayed greater maximum response magnitudes (Top) than *Cntnap2* WT animals (*p* < 0.0001; Fig. 4A, C). Compared to WT rats, *Cntnap2* KO rats also had lower response thresholds (*p* < 0.0001; Fig. 4B, D) and ES50 values (*p* = 0.0023; Fig. 4B, E). Finally, *Cntnap2* KO rats showed higher saturation points than *Cntnap2* WT rats (*p* = 0.0425; Fig. 4B, F). These parameters are indicative of both an upward (startle scaling, as indicated by increased Top) and leftward (sound scaling, indicated by lower threshold, ES50, and saturation point) shifted *Cntnap2* KO baseline startle curve.

### Sound scaling, but not startle scaling, by a prepulse is impacted in *Cntnap2* KO rats

PPI describes the attenuation of startle through a prepulse preceding the startle pulse. The attenuation of startle occurs through both startle and sound scaling. To determine if either scaling component is altered in *Cntnap2* KO animals when a prepulse precedes a startle pulse, parameters extracted from the respective startle response curve fittings were evaluated within genotype. Startle scaling was assessed by examining changes in maximum response magnitude (Top) between startle-alone and prepulse/startle trials. Sound scaling was assessed by comparing changes in response threshold, ES50, and saturation point values between startle-alone and prepulse/startle trials in the normalized curve fitting method as described above.

#### Maximum response magnitude (Top) as a measure of startle scaling

**Fig. 5:**
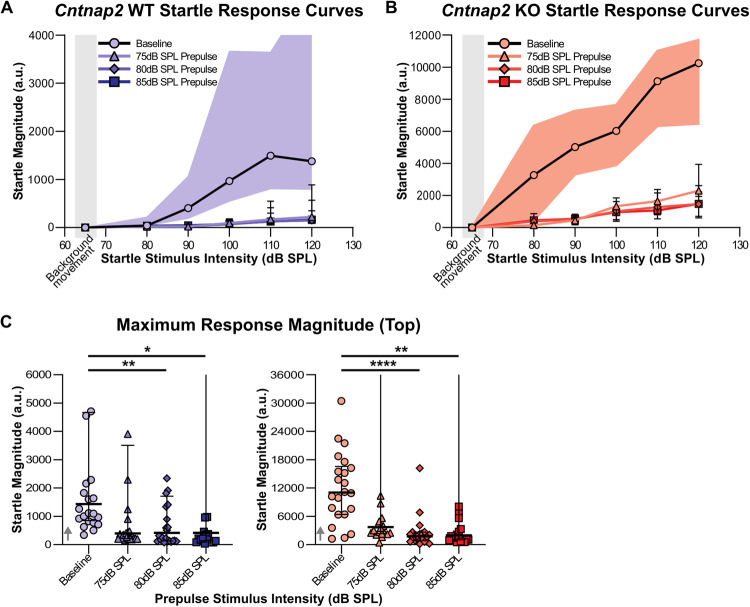
Comparison of startle scaling parameters between baseline (circle) and prepulse conditions (75 dB as triangles, 80 dB as diamonds, and 85 dB as squares) within each genotype. **A** Plots of startle response curves for *Cntnap2* WT rats. Note: Y axis scales for *Cntnap2* WTs and KOs are different as *Cntnap2* KOs have a greater baseline startle response magnitude than WTs. IQR for the baseline response curve is visualized as the shaded area. Goodness of fit Sy.x: Baseline = 2310, 75 dB = 290.4, 80 dB = 218.9, 85 dB = 215.4. **B** Plots of startle response curves for *Cntnap2* KO rats. Goodness of fit Sy.x: Baseline = 3449, 75 dB = 1041, 80 dB = 936, 85 dB = 742.3. **C** Maximum startle response (Top). *Cntnap2* WTs are represented in blue and *Cntnap2* KOs in red. Scatter plots represent individual values and black lines represent median with error bars as IQR. Vertical lines indicate that there are values outside the limits of the y axis, but to visualize the data more clearly, graphs were zoomed in. Both *Cntnap2* WTs and KOs showed a decrease in the maximum startle response magnitude from the baseline startle response curve with the 80 dB and 85 dB prepulse stimuli. There was no difference between the baseline and 75 dB prepulse stimulus. Post hoc: Dunn’s test of multiple comparison; *p < 0.05, **p < 0.01, ****p < 0.0001, no asterisk indicates non-significance of the comparison.

Top values extracted from the startle response curves were compared within genotype to determine if startle scaling occurred (Fig. 5 and Supplementary Table 4). Friedman tests followed by post hoc Dunn’s multiple comparison (baseline vs 75 dB prepulse, baseline vs 80 dB prepulse, and baseline vs 85 dB prepulse trials) revealed that in WT rats, there was an effect of prepulse intensity on Top values (*X*^2^(3) = 11.50, *p* = 0.0093), with post hoc analyses revealing that the 80 dB and 85 dB prepulses resulted in an attenuated maximum startle response (Baseline vs 80 dB: *p* = 0.0052, Baseline vs 85 dB: *p* = 0.0219; Fig. 5A, C; Supplementary Table 4). The Top value with the 75 dB prepulse showed no statistical difference from baseline (Baseline vs 75 dB: *p* = 0.2209; Fig. 5C and Supplementary Table 4). In *Cntnap2* KO rats, there was also an effect of prepulse on Top values (*X*^2^(3) = 23.18, *p* < 0.0001), with post hoc analyses revealing that the 80 dB and 85 dB prepulse stimuli resulted in an attenuated maximum startle response (Baseline vs 80 dB: *p* < 0.0001, Baseline vs 85 dB: *p* = 0.0048; Fig. 5A, C; supplementary Table 4). As in WT animals, Top value with the 75 dB prepulse showed no statistical difference from baseline (Baseline vs 75 dB: *p* = 0.5969; Fig. 5C and Supplementary Table 4). In summary, a significant reduction of maximum startle magnitude by the 80 dB and 85 dB prepulses, indicative of startle scaling, was observed in both genotypes.

#### Threshold, ES50, and saturation point as measures of sound scaling

**Fig. 6:**
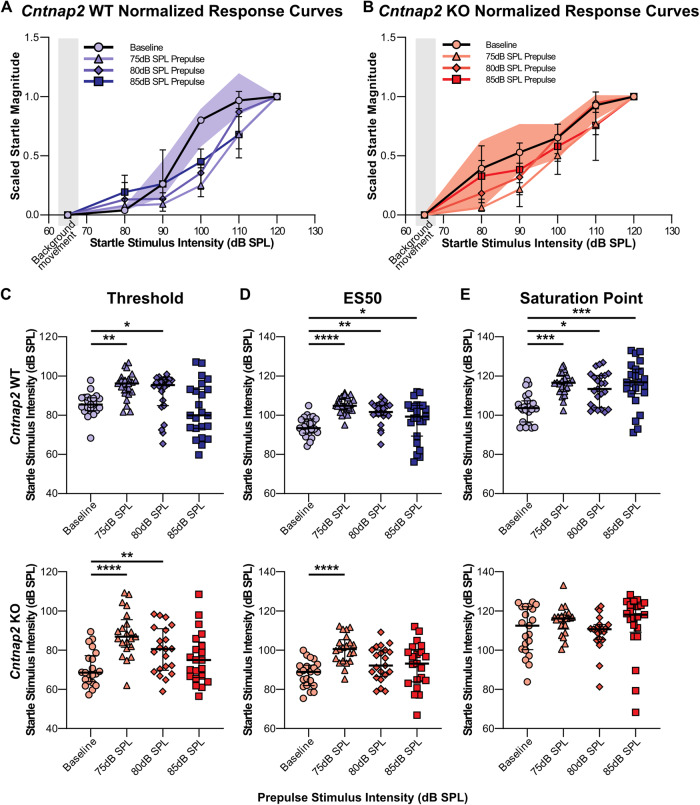
Comparison of sound scaling parameters between baseline (circle) and prepulse conditions (75 dB as triangles, 80 dB as diamonds, and 85 dB as squares) within each genotype. **A** Plots of scaled startle response curves for *Cntnap2* WT. IQR for the baseline response is visualized as the shaded area. Goodness of fit Sy.x: Baseline = 0.1895, 75 dB = 0.1276, 80 dB = 0.1884, 85 dB = 0.2158. **B** Plots of scaled startle response curves for *Cntnap2* KO rats. Goodness of fit Sy.x: Baseline = 0.1823, 75 dB = 0.1736, 80 dB = 0.2029, 85 dB = 0.2433. **C** Threshold (10% of Top). *Cntnap2* WTs are represented in blue and *Cntnap2* KOs in red. Scatter plots represent individual values and black lines represent the median with error bars as IQR. Both *Cntnap2* WTs and KOs showed an increase in threshold from baseline with the 80 dB and 85 dB prepulse stimuli. There was no difference between the baseline and 75 dB prepulse stimulus. **D** ES50 (50% of Top). *Cntnap2* WTs showed an increase in ES50 from baseline at all prepulse intensities, while *Cntnap2* KOs showed an increase in ES50 with the 75 dB prepulse from baseline, but not with the 80 dB or 85 dB prepulse stimuli**. E** Saturation point (90% of Top). *Cntnap2* WTs showed an increase in saturation point from baseline with all prepulse stimuli, while *Cntnap2* KOs showed no differences in this parameter from baseline with any of the prepulse stimuli. Post hoc: Dunn’s test of multiple comparison; *p < 0.05, *p < 0.01, ***p < 0.001, ****p < 0.0001.

To examine differences in threshold, ES50, and saturation point between baseline and prepulse-startle curves within genotype, Friedman tests followed by post hoc Dunn’s multiple comparison were conducted (baseline vs 75 dB prepulse intensity, baseline vs 80 dB prepulse intensity, and baseline vs 85 dB prepulse intensity). An effect of prepulse on threshold was found in WT rats (*X*^2^(3) = 19.43, *p* = 0.0002), with post hoc analyses revealing that the 75 dB and 80 dB prepulse intensities resulted in increased threshold values from baseline (Baseline vs 75 dB: *p* = 0.0061, Baseline vs 80 dB: *p* = 0.0259; Fig. 6C and Supplementary Table 5). No difference was found between the baseline and 85 dB prepulse conditions (Baseline vs 85 dB: *p* > 0.9999). An effect of prepulse on threshold was also found in *Cntnap2* KO rats (*X*^2^(3) = 29.74, *p* < 0.0001), with post hoc analyses revealing that the 75 dB and 80 dB prepulse stimuli resulted in increased threshold from baseline (Baseline vs 75 dB: *p* < 0.0001, Baseline vs 80 dB: *p* = 0.0038). No difference was found between the baseline and 85 dB prepulse conditions (Baseline vs 85 dB: *p* = 0.4545; Fig. 6C and Supplementary Table 5).

An effect of prepulse on ES50 was found in WT rats (*X*^2^(3) = 28.62, *p* < 0.0001), with post hoc analyses revealing that all prepulse stimulus intensities resulted in increased ES50 values from baseline (Baseline vs 75 dB: *p* < 0.0001, Baseline vs 80 dB: *p* = 0.0012, Baseline vs 85 dB: *p* = 0.0422; Fig. 6D and Supplementary Table 6). In *Cntnap2* KO rats, an effect of prepulse on ES50 was also found (*X*^2^(3) = 27.46, *p* < 0.0001), with post hoc analyses revealing a significant difference between the baseline and 75 dB prepulse curves only (Baseline vs 75 dB: *p* < 0.0001). However, no differences in ES50 were found between the baseline condition and 80 dB or 85 dB prepulse stimuli (Baseline vs 80 dB: *p* = 0.219, Baseline vs 85 dB: *p* = 0.1675; Fig. 6D and Supplementary Table 6).

Finally, an effect of prepulse on saturation point was found in WT rats (*X*^2^(3) = 19.64, *p* = 0.0002), with post hoc analyses revealing that all prepulse stimuli resulted in increased saturation points from baseline (Baseline vs 75 dB: *p* = 0.0002, Baseline vs 80 dB: *p* = 0.0259, Baseline vs 85 dB: *p* = 0.0008; Fig. 6E and Supplementary Table 6). In contrast, no effect of prepulse on saturation point was found in *Cntnap2* KO rats (*X*^2^(3) = 4.600, *p* = 0.2035).

In summary, an increase in all three parameters (threshold, ES50, and saturation point) in the WT animals documents a right shift of the startle response curve, confirming the presence of sound scaling by the prepulse. In *Cntnap2* KO animals, however, this right shift is present for the threshold only, but not for the saturation point and only partly for the ES50. This indicates that the startle response curve is only shifted at low startle intensities, but not at higher startle intensities. In other words, at louder startle sounds, which are commonly used in classical startle testing, sound scaling is deficient in *Cntnap2* KO rats.

Overall, *Cntnap2* KO rats show increased baseline startle due to an upwards and leftward shift of the startle i/o function, and independently from that, a disruption in PPI that is due to deficient sound scaling, while startle scaling by the prepulse is intact.

## Discussion

The present study investigated changes in startle and PPI in *Cntnap2* KO rats, using an advanced PPI assessment method, partly based on Miller et al. [12]. The results of this study suggest that altered PPI in *Cntnap2* KO rats is caused by a partial disruption in sound scaling, while startle scaling remains intact. These results are not only consistent with previous findings of perturbed sensorimotor gating in this highly translational model [28–30], but extend and refine these findings by providing some indication for potentially affected neural pathways and mechanisms.

### Addressing the assumption of normality

A primary assumption in startle studies is that startle responses repeatedly measured within an animal have a Gaussian distribution. This leads to the use of parametric statistics, which are inappropriate when data is in violation of normality [34]. Miller et al. [12] proposed to use of a log_10_ transformation to improve startle data normality. While we found the majority of within-animal startle response distributions to be skewed, non-normal distribution was evident mostly for low startle stimulus intensities or startle trials with a prepulse, when the resulting startle response magnitude was very low. In contrast, baseline startle (startle-alone) data was more often normally distributed and lost normality when log_10_ transformed. This observation is critical, as the baseline startle response is always utilized as the reference in the quantification of % PPI (Eq. 1 [9, 35]). Log_10_ transforming baseline startle data might therefore introduce a systemic bias when calculating PPI. Furthermore, given that most startle responses were not normally distributed, we used non-parametric tests for analyses, as these are not based on the assumption of normality. Ignoring the assumptions of statistical tests puts studies at risk of error [34], we, therefore, propose to not log_10_ transform startle data, but to use non-parametric data analyses and statistics for startle and PPI analysis.

### Altered baseline startle in *Cntnap2* KO rats

The *Cntnap2* KO rat model has a high face and constructs validity for core autism-related alterations in social and stereotypic behaviors, as well as in sensory filtering and sensorimotor gating [3, 28–31]. Our advanced analyses demonstrate that the baseline startle response curve is scaled in both the leftward and upward directions in *Cntnap2* KO rats, which confirms and expands on previous studies reporting higher startle responses in these rats [28, 30]. As the baseline startle response is determined by PnC giant neuronal activity [7, 24, 26], the hyperreactivity to sound could be a result of hyperexcitability of PnC giant neurons or of altered excitatory glutamatergic input [18]. Scott et al. [29] found no differences in auditory brainstem responses between adult *Cntnap2* WT and KO rats, indicating that there are no changes in sound processing at the level of the auditory nerve, or cochlear nucleus. However, an imbalance in excitatory (glutamate) and inhibitory (GABA) neurotransmitters has been shown in *Cntnap2* KO rats, with higher levels of excitatory neurotransmitters in the PnC, which may be responsible for their increased responsivity to sound [28]. Indeed, in vivo, electrophysiological studies in the PnC of *Cntnap2* KO rats showed increased excitability of startle-mediating PnC giant neurons in response to sound [36]. Interestingly, this was mostly true for only females, indicating that in males alternative strategies, e.g. the recruitment of more PnC giant neurons at a given sound intensity, may instead lead to higher startle.

### Altered sensorimotor gating in *Cntnap2* KO rats

Consistent with previous reports we found deficient %PPI in *Cntnap2* KO rats [28–30], specifically at the 100 dB startle stimulus intensity. In a study by Möhrle and colleagues [28], *Cntnap2* WT response thresholds and saturation points were determined to be 86.6 dB and 106.0 dB respectively; similarly, this study found *Cntnap2* WT response thresholds and saturation points to be 85.41 dB and 103.6 dB, respectively. As modulation of the startle response by a prepulse is most likely to occur within this range [10, 11, 25], a deficit in PPI would also be most evident at these stimulus intensities. Indeed, a PPI deficit at all prepulse intensities was found at the 100 dB startle stimulus, and to a lesser extent at the 110 dB startle stimulus intensity.

### Sound scaling, but not startle scaling, is specifically affected in *Cntnap2* KO rats

As startle scaling represents a change in reflex capacity, it is suggested that this component relates to the motor output and thus the primary startle pathway, specifically at the level of the PnC [11]. Giant neurons in the PnC receive sensory input from the cochlear root nucleus and provide output to motor neurons [7, 26]. Martin-Iverson and Stevenson [11] described reflex capacity as the maximum amount of startle physically possible and this may represent the maximum neural response in pre-motor neurons in the PnC. Both genotypes exhibit evidence of startle scaling during PPI through changes in maximum startle response magnitudes, which suggests that although there are differences in baseline startle, the differences in PPI between the genotypes are likely not due to changes at the level of the PnC or primary startle pathway.

*Cntnap2* KO rats showed increased threshold values from baseline, however, a right shift was not consistent across the other two measures of sound scaling, ES50 and saturation points, indicating disruptions in sound scaling. This suggests the involvement of midbrain structures (i.e., pedunculopontine tegmental nucleus, PPTg, Supplemental Fig. 1) and/or changes to top–down modulatory input in *Cntnap2* KO rats, as sound scaling represents a change in sound processing mechanisms due to the prepulse. Several studies examine the effect of loss of *Cntnap2* on higher-order auditory structures, which may influence sensorimotor gating mechanisms. Changes in cortical GABAergic and inhibitory interneurons have been found in the *Cntnap2* KO mouse [37], supporting a general excitatory-inhibitory imbalance potentially playing a role in the PPI deficit. Indeed, previous work, ranging from in vitro electrophysiology [38] to human brain imaging [39], has demonstrated neuronal and network-level cortical alterations in rodent models and humans with *Cntnap2* mutation. Electrophysiological recordings of auditory cortical neurons with *Cntnap2* KO showed hyperexcitability and longer firing rates in pyramidal neurons than in *Cntnap2* WTs [38]. Additionally, histological analysis of the *Cntnap2* KO mouse has shown neuronal migration errors in cortical layers, in combination with decreased dendritic spine development and arborization of pyramidal neurons, indicating that loss of the *Cntnap2* gene influences structures and pathways relevant for sound processing ([37, 40], see also [41]). Furthermore, mass-spectrometry quantification of neurotransmitters in the brainstem showed that inhibitory GABA neurotransmitter levels in the PnC are altered in *Cntnap2* KO rats [28]. GABAergic input from the PPTg to the PnC has been suggested to mediate at least a portion of PPI [17, 18], so neurotransmitter imbalance at this connection may play a role in the prepulse processing alterations. Truong et al. [42] also found reduced GABAergic interneurons in the medial geniculate nucleus in *Cntnap2* KO animals, which may also result in reduced response inhibition. Taken together, molecular, histological, neural, and electrophysiological studies along with the behavioral scaling analysis suggest a potential influence of higher-order structures on altered sound processing in *Cntnap2* KO rats. Future research should focus on the potential role of these higher-order alterations in the sound scaling of startle.

### Conclusion

Overall, this study used a novel and more detailed method to assess startle and PPI in *Cntnap2* KO rats. The results confirm that startle and sound scaling by a prepulse are two independent processes, as only sound scaling, but not startle scaling, is altered in *Cntnap2* KO rats, leading to the reported PPI deficit. This supports a potential role of midbrain and/or higher-order sound processing structures in altered sensorimotor gating. Our results suggest that for future assessment of PPI in preclinical models, startle stimuli should encapsulate the whole range of the i/o startle curve, including the response threshold, the dynamic range, and the saturation point, without exceeding the known auditory pain threshold. Considerations should also be given to the distribution of data, as normality should not be assumed for startle data.

### Supplementary information

Supplementary Material

## References

[1] Takahashi H, Nakahachi T, Komatsu S, Ogino K, Iida Y, Kamio Y (2014). Hyperreactivity to weak acoustic stimuli and prolonged acoustic startle latency in children with autism spectrum disorders. Mol Autism.

[2] Takahashi H, Kamio Y (2018). Acoustic startle response and its modulation in schizophrenia and autism spectrum disorder in Asian subjects. Schizophr Res.

[3] Möhrle D, Fernández M, Peñagarikano O, Frick A, Allman B, Schmid S (2020). What we can learn from a genetic rodent model about autism. Neurosci Biobehav Rev.

[4] Koch M (1999). The neurobiology of startle. Prog Neurobiol.

[5] Gomez-Nieto R, Hormigo S, Lopez DE (2020). Prepulse inhibition of the auditory startle reflex assessment as a hallmark of brainstem sensorimotor gating mechanisms. Brain Sci.

[6] Bowen GP, Lin D, Taylor MK, Ison JR (2003). Auditory cortex lesions in the rat impair both temporal acuity and noise increment thresholds, revealing a common neural substrate. Cereb Cortex.

[7] Koch M, Lingenhohl K, Pilz PKD (1992). Loss of the acoustic startle response following neurotoxic lesions of the caudal pontine reticular formation: Possible role of giant neurons. Neuroscience.

[8] Ison JR, O’Connor K, Bowen GP, Bocirnea A (1991). Temporal resolution of gaps in noise by the rat is lost with functional decortication. Behav Neurosci.

[9] Csomor PA, Yee BK, Vollenweider FX, Feldon J, Nicolet T, Quednow BB (2008). On the influence of baseline startle reactivity on the indexation of prepulse inhibition. Behav Neurosci.

[10] Csomor PA, Yee BK, Quednow BB, Stadler RR, Feldon J, Vollenweider FX (2006). The monotonic dependency of prepulse inhibition of the acoustic startle reflex on the intensity of the startle-eliciting stimulus. Behav Brain Res.

[11] Martin-Iverson MT, Stevenson KN (2005). Apomorphine effects on emotional modulation of the startle reflex in rats. Psychopharmacol (Berl).

[12] Miller EA, Kastner DB, Grzybowski MN, Dwinell MR, Geurts AM, Frank LM (2020). Robust and replicable measurement for prepulse inhibition of the acoustic startle response. Mol Psychiatry.

[13] Azzopardi E, Louttit AG, DeOliveira C, Laviolette SR, Schmid S (2018). The role of cholinergic midbrain neurons in startle and prepulse inhibition. J Neurosci.

[14] Weible AP, Yavorska I, Kayal D, Duckler U, Wehr M (2020). A layer 3→5 circuit in auditory cortex that contributes to pre-pulse inhibition of the acoustic startle response. Front Neural Circuits.

[15] Cano JC, Huang W, Fénelon K (2021). The amygdala modulates prepulse inhibition of the auditory startle reflex through excitatory inputs to the caudal pontine reticular nucleus. BMC Biol.

[16] Weible AP, Yavorska I, Wehr M (2020). A cortico-collicular amplification mechanism for gap detection. Cereb Cortex.

[17] Fulcher N, Azzopardi E, De Oliveira C, Hudson R, Schormans AL, Zaman T (2020). Deciphering midbrain mechanisms underlying prepulse inhibition of startle. Prog Neurobiol.

[18] Yeomans JS, Bosch D, Alves N, Daros A, Ure RJ, Schmid S (2010). GABA receptors and prepulse inhibition of acoustic startle in mice and rats. Eur J Neurosci.

[19] Brosda J, Hayn L, Klein C, Koch M, Meyer C, Schallhorn R (2011). Pharmacological and parametrical investigation of prepulse inhibition of startle and prepulse elicited reactions in Wistar rats. Pharm Biochem Behav.

[20] Geis HR, Schmid S (2011). Glycine inhibits startle-mediating neurons in the caudal pontine reticular formation but is not involved in synaptic depression underlying short-term habituation of startle. Neurosci Res.

[21] Ison JR, Bowen GP (2000). Scopolamine reduces sensitivity to auditory gaps in the rat, suggesting a cholinergic contribution to temporal acuity. Hear Res.

[22] Stanley-Cary CC, Harris C, Martin-Iverson MT (2002). Differing effects of the cannabinoid agonist, CP 55,940, in an alcohol or Tween 80 solvent, on prepulse inhibition of the acoustic startle reflex in the rat. Behav Pharm.

[23] Yeomans JS, Lee J, Yeomans MH, Steidl S, Li L (2006). Midbrain pathways for prepulse inhibition and startle activation in rat. Neuroscience.

[24] Zheng A, Schmid S (2023). A review of the neural basis underlying the acoustic startle response with a focus on recent developments in mammals. Neurosci Biobehav Rev.

[25] Hince DA, Martin-Iverson MT (2005). Differences in prepulse inhibition (PPI) between Wistar and Sprague-Dawley rats clarified by a new method of PPI standardization. Behav Neurosci.

[26] Lee Y, Lopez DE, Meloni EG, Davis’ M (1996). A primary acoustic startle pathway: Obligatory role of cochlear root neurons and the nucleus reticularis pontis caudalis. J Neurosci.

[27] Valsamis B, Schmid S (2011). Habituation and prepulse inhibition of acoustic startle in rodents. J Vis Exp.

[28] Möhrle D, Wang W, Whitehead SN, Schmid S (2021). GABA B receptor agonist R-Baclofen reverses altered auditory reactivity and filtering in the Cntnap2 knock-out rat. Front Integr Neurosci.

[29] Scott KE, Schormans XAL, Pacoli KY, De Oliveira C, Allman BL, Schmid S (2018). Altered auditory processing, filtering, and reactivity in the Cntnap2 knock-out rat model for neurodevelopmental disorders. J Neurosci.

[30] Scott KE, Kazazian K, Mann RS, Möhrle D, Schormans AL, Schmid S (2020). Loss of Cntnap2 in the rat causes autism-related alterations in social interactions, stereotypic behavior, and sensory processing. Autism Res.

[31] Thomas AM, Schwartz MD, Saxe MD, Kilduff TS (2017). Cntnap2 knockout rats and mice exhibit epileptiform activity and abnormal sleep—wake physiology. Sleep.

[32] Strauss KA, Puffenberger EG, Huentelman MJ, Gottlieb S, Dobrin SE, Parod JM (2006). Recessive symptomatic focal epilepsy and mutant contactin-associated protein-like 2. N Engl J Med.

[33] Altman DG, Bland JM (1995). The normal distribution. BMJ.

[34] Baker D, Lidster K, Sottomayor A, Amor S (2014). Two years later: journals are not yet enforcing the ARRIVE guidelines on reporting standards for pre-clinical animal studies. PLoS Biol.

[35] Blumenthal TD, Elden A, Flaten MA (2004). A comparison of several methods used to quantify prepulse inhibition of eyeblink responding. Psychophysiology.

[36] Zheng A, Scott KE, Schormans AL, Mann R, Allman BL, Schmid S (2023). Differences in startle and prepulse inhibition in contactin-associated protein-like 2 knock-out rats are associated with sex-specific alterations in brainstem neural activity. Neuroscience.

[37] Penagarikano O, Abrahams BS, Herman EI, Winden KD, Gdalyahu A, Dong H (2011). Absence of CNTNAP2 leads to epilepsy, neuronal migration abnormalities, and core autism-related deficits. Cell.

[38] Scott KE, Mann RS, Schormans AL, Schmid S, Allman BL. Hyperexcitable and immature-like neuronal activity in the auditory cortex of adult rats lacking the language-linked CNTNAP2 gene. Cereb Cortex. 2022;32:4797–817. 10.1093/cercor/bhab517 PMC9626820 35106542

[39] Scott-Van Zeeland AA, Abrahams BS, Alvarez-Retuerto AI, Sonnenblick LI, Rudie JD, Ghahremani D (2010). Altered functional connectivity in frontal lobe circuits is associated with variation in the autism risk gene CNTNAP. Sci Transl Med.

[40] Anderson GR, Galfin T, Xu W, Aoto J, Malenka RC, Südhof TC (2012). Candidate autism gene screen identifies critical role for cell-adhesion molecule CASPR2 in dendritic arborization and spine development. Proc Natl Acad Sci USA.

[41] Varea O, Martin-de-Saavedra MD, Kopeikina KJ, Schürmann B, Fleming HJ, Fawcett-Patel JM (2015). Synaptic abnormalities and cytoplasmic glutamate receptor aggregates in contactin associated protein-like 2/Caspr2 knockout neurons. Proc Natl Acad Sci USA.

[42] Truong DT, Rendall AR, Castelluccio BC, Eigsti IM, Fitch HR (2015). Auditory processing and morphological anomalies in medial geniculate nucleus of Cntnap2 mutant mice. Behav Neurosci.

