# Supplementary material for: Assessing the Cntnap2 knockout rat prepulse inhibition deficit through prepulse scaling of the baseline startle response curve
Source: Transl Psychiatry. 2023 Oct 18;13:321. doi: 10.1038/s41398-023-02629-6 (PMC10584930; doi:10.1038/s41398-023-02629-6)
Supplement: Supplementary file 1 — Supplementary Material [file 41398_2023_2629_MOESM1_ESM.docx]

**
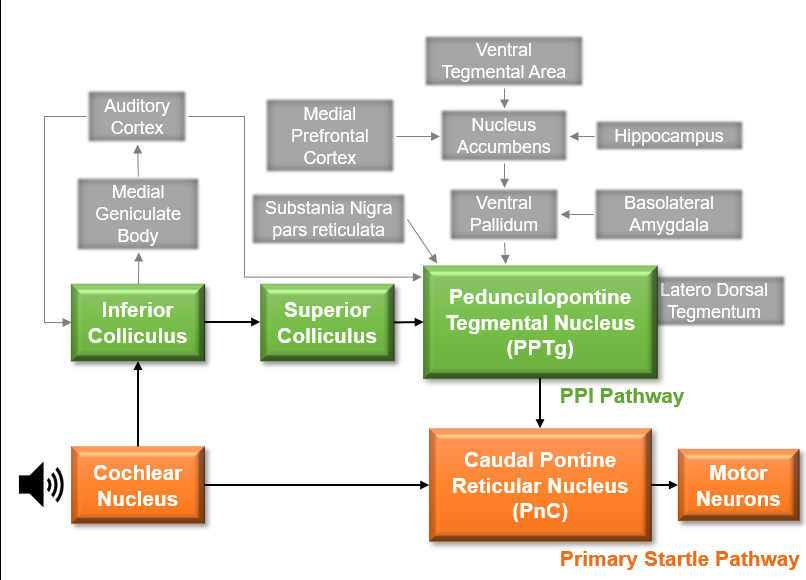
**

**Supplementary Figure 1.** Simplified scheme of the primary startle (orange) and PPI (green) pathways. The primary startle pathway involves three serially connecting structures in the brainstem: the cochlear nucleus, caudal pontine reticular nucleus (PnC), and motor neurons. PPI is mediated by feedforward mechanisms from midbrain and higher order structures; the midbrain pathway involves the inferior colliculus (IC), superior colliculus (SC), and the pedunculopontine tegmental nucleus (PPTg). The PPTg provides inhibitory input to the PnC leading to a decreased startle response. Pathway figure developed based on the reviews and findings of Koch (1), Gomez-Nieto et al. (2), Fulcher et al. (3), and Weible et al. (4).

**
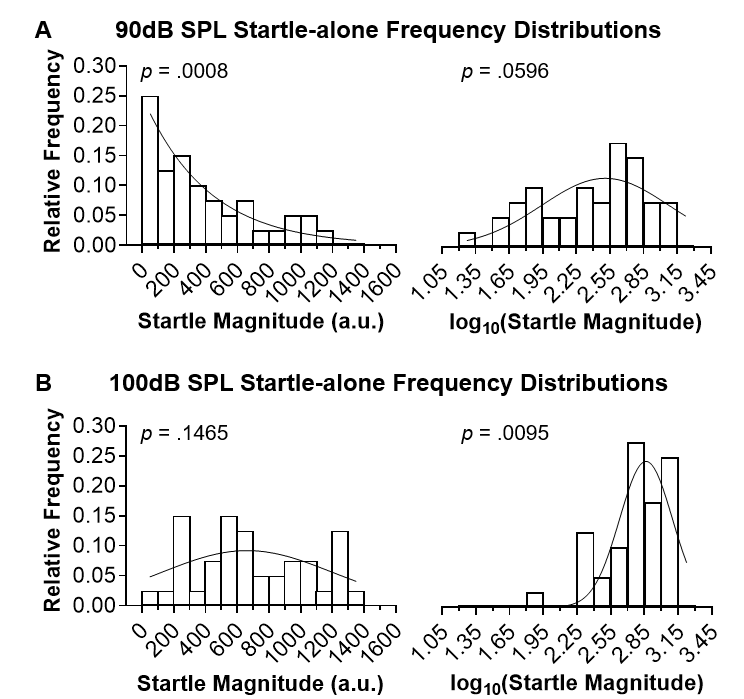
**

**Supplementary Figure 2.** Frequency distributions of startle magnitudes from a sample animal (*Cntnap2* WT M) at 90dB and 100dB startle-alone conditions. Normality of the responses was assessed using the Shapiro-Wilk test and histograms were created using all startle responses to the stimulus intensity of interest. **A)** The 90dB startle-alone histogram shows a non-Gaussian distribution with the raw startle magnitudes and a Gaussian distribution with the log10 transformation. **B)** The 100dB startle-alone histograms show normality of the distribution with the raw startle magnitudes and the loss of normality with the log10 transformation.

**
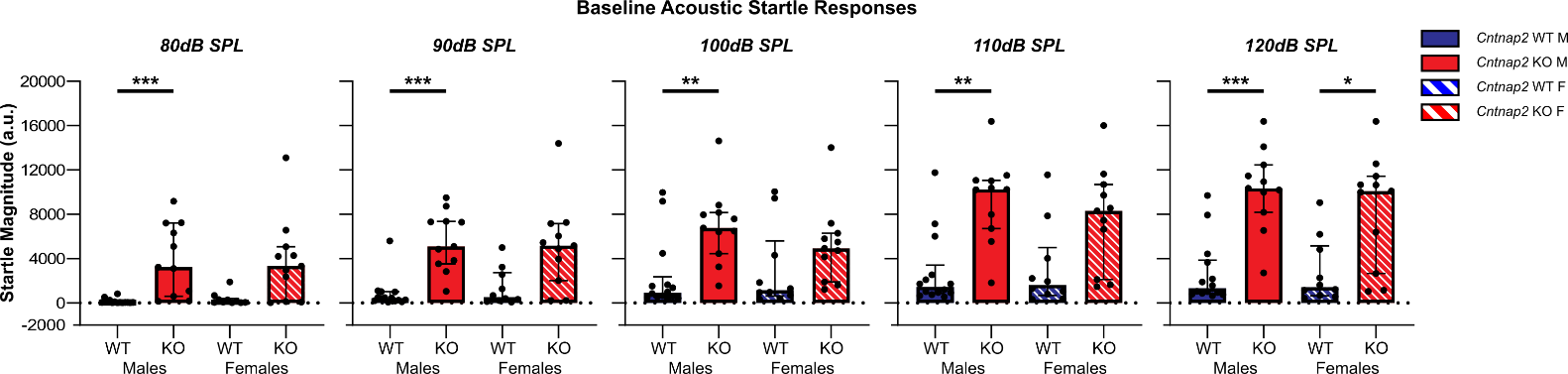
**

**Supplementary Figure 3**. Classical assessment of startle. *Cntnap2* WTs are represented in blue and *Cntnap2* KOs in red; males are represented by solid bars and females by diagonal bars. Scatter plots represent individual animals and bars represent group medians with error bars as IQR. Baseline acoustic startle response magnitudes for startle alone conditions (from left to right: 80dB, 90dB, 100dB, 110dB and 120dB) were compared using Kruskal-Wallis test followed by Dunn’s test of multiple comparisons for relevant comparisons (i.e., *Cntnap2* WT M vs *Cntnap2* KO M, *Cntnap2* WT M vs *Cntnap2* WT F, *Cntnap2* WT F vs *Cntnap2* KO F, *Cntnap2* KO M vs *Cntnap2* KO F). At the 80dB (*p* = 0.0004), 90dB (*p* =0.0004), 100dB (*p* = 0.0037), and 110dB (*p* = 0.0025) startle stimulus intensities *Cntnap2* KO M had greater baseline startle responses than *Cntnap2* WT M. At the 120dB startle stimulus intensity, *Cntnap2* KO M had a greater baseline startle response magnitude than *Cntnap2* WT M (*p* = 0.0004) and *Cntnap2* KO F had a greater baseline startle response magnitude than *Cntnap2* WT F (*p* = 0.239). ^*^*p* <0.05, ^**^*p* <0.01, ^***^*p* <0.001, no asterisk indicates non-significance of the comparison.


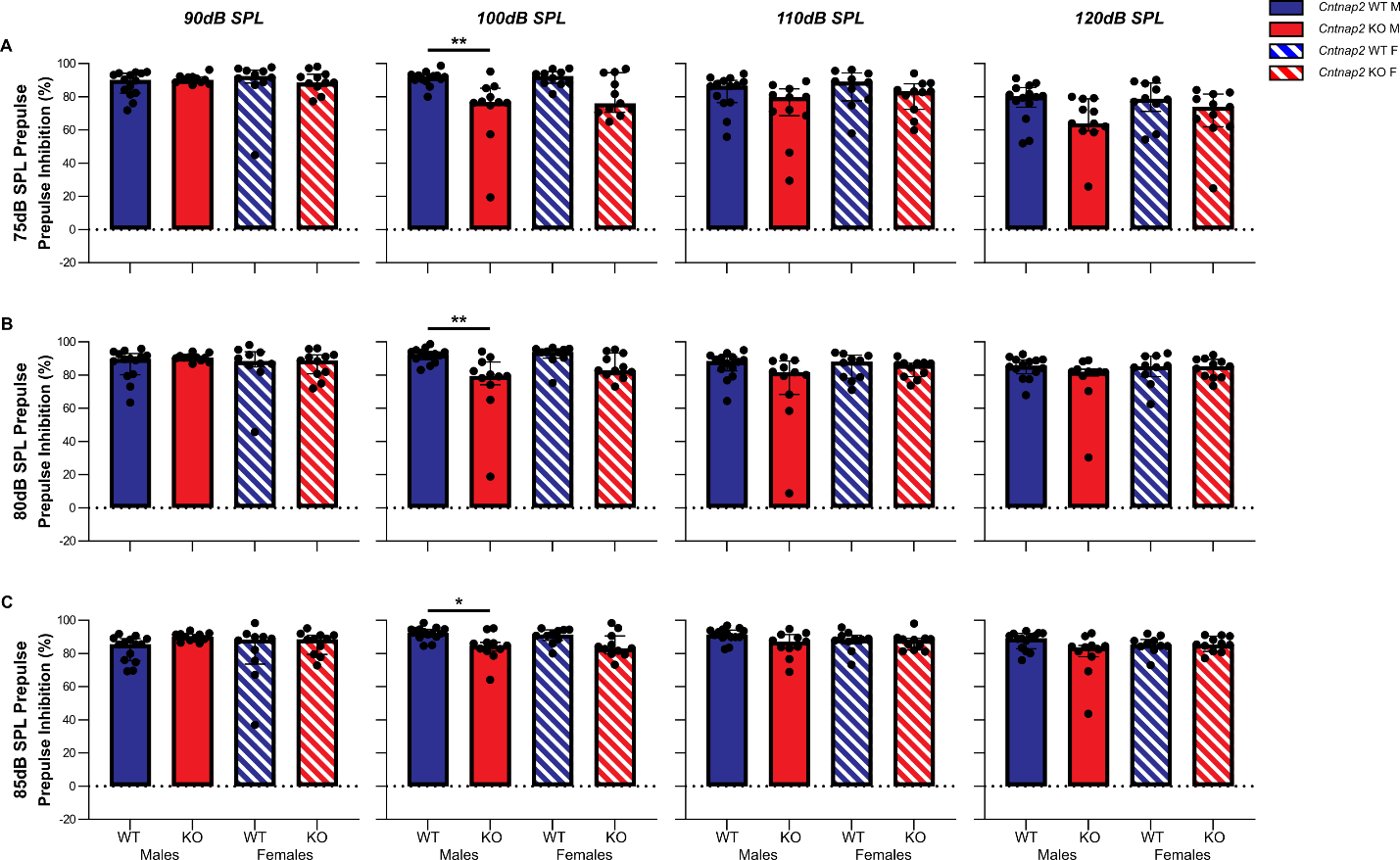


**Supplementary Figure 4.** Classical assessment of PPI split by sex. *Cntnap2* WTs are represented in blue and *Cntnap2* KOs in red; males are represented by solid bars and females by diagonal bars. Scatter plots represent individual animals and bars represent group medians with error bars as IQR. Startle stimulus intensities are presented from left to right in increasing order (90dB, 100dB, 110dB and 120dB) with the three prepulse intensities (top to bottom: 75dB, 80dB, and 85dB). %PPI for each prepulse and startle stimulus condition was compared using Kruskal-Wallis test followed by Dunn’s test of multiple comparisons for relevant comparisons (i.e., *Cntnap2* WT M vs *Cntnap2* KO M, *Cntnap2* WT M vs *Cntnap2* WT F, *Cntnap2* WT F vs *Cntnap2* KO F, *Cntnap2* KO M vs *Cntnap2* KO F). **A)** 75dB prepulse. *Cntnap2* KO M had lower %PPI compared to *Cntnap2* WT M at the 100dB startle stimulus intensity (*p* = 0.0096). **B)** 80dB prepulse. *Cntnap2* KO M had lower %PPI compared to *Cntnap2* WT M at the 100dB startle stimulus intensity (*p* = 0.0057). **C)** 85dB prepulse. *Cntnap2* KO M had lower %PPI compared to *Cntnap2* WT M at the 100dB startle stimulus intensity (*p* = 0.04). Despite the Kruskal-Wallis test showing significance at the 110dB startle stimulus intensity, X^2^(3) = 8.7, *p* = 0.0336, *post-hoc* tests revealed no differences between any of the comparisons. ^*^*p* <0.05, ^**^*p* <0.01, no asterisk indicates non-significance of the comparison.

| **Supplementary Table 1.** Proportion of population with normally distributed startle responses measured at the individual animal level. | | | | | | | | | | | |
| --- | --- | --- | --- | --- | --- | --- | --- | --- | --- | --- | --- |
|  |  | **Peak-to-peak raw data** | | | | | **log of peak-to-peak** | | | | |
| **Startle Stimulus Intensity (dB SPL)** | **Prepulse Intensity (dB SPL)** | **WT M** | **KO M** | **WT F** | **KO F** | **% of total population** | **WT M** | **KO M** | **WT F** | **KO F** | **% of total population** |
| 80dB | Baseline | 0 | 1 | 0 | 1 | 4.35% | 7 | 2 | 6 | 0 | 32.61% |
|  | 75dB | 0 | 0 | 0 | 0 | 0.00% | 4 | 4 | 4 | 7 | 41.30% |
|  | 80dB | 0 | 0 | 0 | 0 | 0.00% | 8 | 7 | 6 | 5 | 56.52% |
|  | 85dB | 0 | 1 | 0 | 1 | 4.35% | 6 | 3 | 5 | 5 | 41.30% |
| 90dB | Baseline | 0 | 3 | 1 | 4 | 17.39% | 7 | 0 | 6 | 2 | 32.61% |
|  | 75dB | 0 | 0 | 0 | 0 | 0.00% | 3 | 7 | 4 | 6 | 43.48% |
|  | 80dB | 0 | 0 | 0 | 0 | 0.00% | 4 | 10 | 5 | 5 | 52.17% |
|  | 85dB | 0 | 0 | 0 | 0 | 0.00% | 9 | 6 | 5 | 4 | 52.17% |
| 100dB | Baseline | 4 | 7 | 5 | 8 | **52.17%** | 1 | 1 | 2 | 0 | **8.70%** |
|  | 75dB | 0 | 1 | 0 | 1 | 4.35% | 10 | 7 | 5 | 8 | 65.22% |
|  | 80dB | 0 | 0 | 0 | 0 | 0.00% | 8 | 3 | 4 | 8 | 50.00% |
|  | 85dB | 0 | 0 | 0 | 2 | 4.35% | 6 | 7 | 7 | 6 | 56.52% |
| 110dB | Baseline | 6 | 3 | 6 | 3 | **39.13%** | 3 | 1 | 4 | 0 | **17.39%** |
|  | 75dB | 0 | 0 | 0 | 1 | 2.17% | 12 | 6 | 5 | 10 | 71.74% |
|  | 80dB | 0 | 1 | 0 | 1 | 4.35% | 10 | 7 | 10 | 10 | 80.43% |
|  | 85dB | 0 | 1 | 0 | 0 | 2.17% | 8 | 6 | 8 | 7 | 63.04% |
| 120dB | Baseline | 4 | 2 | 5 | 4 | **32.61%** | 7 | 0 | 6 | 1 | **30.43%** |
|  | 75dB | 2 | 1 | 0 | 2 | 10.87% | 9 | 5 | 8 | 8 | 65.22% |
|  | 80dB | 0 | 2 | 0 | 0 | 4.35% | 12 | 7 | 7 | 10 | 78.26% |
|  | 85dB | 0 | 1 | 0 | 1 | 4.35% | 10 | 5 | 8 | 9 | 69.57% |
| Values in cells are counts of the number of animals from each group whose individual trial distributions meet the Shapiro-Wilk test of normality conditions for a non-significant value and thus are said to have normal distributions. The *% of total population* represents the counts summed together divided by the total population (N = 46). Cells in green highlight the conditions at which a greater proportion of the population had normal distributions when compared to the opposing calculation method. | | | | | | | | | | | |

| **Supplementary Table 2.** Comparison of baseline startle response magnitudes between genotypes. | | | | | |
| --- | --- | --- | --- | --- | --- |
| **Startle Stimulus Intensity (dB SPL)** | **80dB** | **90dB** | **100dB** | **110dB** | **120dB** |
| *Cntnap2* WT | 55 (39.5-261.5) | 417.7 (206.8-1083) | 981.5 (557-3681) | 1505 (820-3657) | 1390 (804.5-4232) |
| *Cntnap2* KO | 3290 (490.3-6374) | 5134 (3356-7291) | 6044 (3923-7669) | 9138 (6362-11024) | 10272 (6509-11713) |
| Mann-Whitney test WT vs KO | < 0.0001^****^ | < 0.0001^****^ | 0.0002^***^ | < 0.0001^****^ | < 0.0001^****^ |
| Median (IQR). Adjusted *p* value using Holm-Sidak correction for multiple comparisons. ^***^*p* < 0.001, ^****^*p* < 0.0001. | | | | | |

| **Supplementary Table 3.** Comparison of %PPI between genotypes. | | | | |
| --- | --- | --- | --- | --- |
|  |  | **Prepulse Intensity (dB SPL)** | | |
| **Startle Stimulus Intensity (dB SPL)** | **Comparison** | **75dB SPL** | **80dB** | **85dB** |
| **90dB SPL** | Wildtypes | 91.3 (84.72-94.65) | 89.79 (82.89-93.43) | 86.06 (75.79-89.73) |
|  | Knockouts | 90.16 (88.05-92.05) | 90.53 (87.45-91.73) | 88.88 (86.4-91.51) |
|  | Mann-Whitney test WT vs. KO | 0.9465^ns^ | 0.9465^ns^ | 0.1123^ns^ |
| **100dB SPL** | Wildtypes | 91.91 (88.8-93.91) | 93.69 (90.03-95.23) | 92.04 (89.72-94.42) |
|  | Knockouts | 76.54 (72.1-86.5) | 82.16 (78.26-89.91) | 83.56 (80.99-87.61) |
|  | Mann-Whitney test WT vs. KO | 0.0002^***^ | 0.0002^***^ | 0.0009^***^ |
| **110dB SPL** | Wildtypes | 87.33 (77.03-91.13) | 88.4 (79.02-91.5) | 89.91 (88.01-93.45) |
|  | Knockouts | 80.92 (70.56-85.28) | 83.27 (78.5-87.28) | 87.5 (82.8-89.47) |
|  | Mann-Whitney test WT vs. KO | 0.0535^(*)^ | 0.0535^(*)^ | 0.0535^(*)^ |
| **120dB SPL** | Wildtypes | 79.58 (75.87-86.36) | 85.36 (80.81-89.33) | 87.65 (83.6-90.77) |
|  | Knockouts | 70.1 (61.79-78.86) | 82.11 (79.24-88.05) | 84.51 (81.06-88.83) |
|  | Mann-Whitney test WT vs. KO | 0.0301^*^ | 0.2163^ns^ | 0.1848^ns^ |
| Median (IQR). Adjusted *p* value using Holm-Sidak correction for multiple comparisons. ^*^*p* < 0.05, ^***^*p* < 0.001, ns: not significant, ^(*)^ trend towards significance. | | | | |

| **Supplementary Table 4.** Within genotype comparison of the startle scaling parameter, maximum response magnitude. | | | |
| --- | --- | --- | --- |
| **Cohort** | **Comparison** | **Maximum response magnitude (Top; a.u.)** | **Baseline vs Prepulse** |
| *Cntnap2* WT | Baseline | 1432 (850.5-4670) | ̶ |
|  | 75dB prepulse | 395.4 (211.3-3505) | 0.2209^ns^ |
|  | 80dB prepulse | 414.2 (134.4-1714) | 0.0052^**^ |
|  | 85dB prepulse | 419.1 (142.3-501925) | 0.0219^*^ |
| *Cntnap2* KO | Baseline | 11039 (6398-16581) | ̶ |
|  | 75dB prepulse | 3735 (2359-333179) | 0.5969^ns^ |
|  | 80dB prepulse | 1751 (825.7-2681) | < 0.0001^****^ |
|  | 85dB prepulse | 1960 (1023-47474) | 0.0048^**^ |
| Median (IQR). Post-hoc: Dunn’s test of multiple comparisons. ^*^*p* < 0.05, ^**^*p* < 0.01, ^***^*p* < 0.001, ^****^*p* < 0.0001, ns: not significant | | | |

| **Supplementary Table 5.** Within genotype comparison of the sound scaling parameter, threshold. | | | |
| --- | --- | --- | --- |
| **Cohort** | **Comparison** | **Threshold (dB SPL)** | **Baseline vs Prepulse** |
| *Cntnap2* WT | Baseline | 85.41 (82.39-88.83), | ̶ |
|  | 75dB prepulse | 96.15 (91.26-98.45) | 0.0061^**^ |
|  | 80dB prepulse | 95.38 (84.88-98.12) | 0.0259^*^ |
|  | 85dB prepulse | 79.94 (73.34-93.22) | > 0.9999^ns^ |
| *Cntnap2* KO | Baseline | 68.55 (63.88-77.17) | ̶ |
|  | 75dB prepulse | 86.91 (81.44-95.58) | < 0.0001^****^ |
|  | 80dB prepulse | 80.50 (69.67-90.90) | 0.0038^**^ |
|  | 85dB prepulse | 74.91 (66.02-84.04) | 0.4545^ns^ |
| Median (IQR). Post-hoc: Dunn’s test of multiple comparisons. ^*^*p* < 0.05, ^**^*p* < 0.01, ^***^*p* < 0.001, ^****^*p* < 0.0001, ns: not significant. | | | |

| **Supplementary Table 6.** Within genotype comparison of the sound scaling parameter, ES50. | | | |
| --- | --- | --- | --- |
| **Cohort** | **Comparison** | **ES50 (dB SPL)** | **Baseline vs Prepulse** |
| *Cntnap2* WT | Baseline | 93.43 (90.84-98.10) | ̶ |
|  | 75dB prepulse | 104.8 (102.5-108.5) | < 0.0001^***^ |
|  | 80dB prepulse | 101.6 (99.75-105.1) | 0.0012^**^ |
|  | 85dB prepulse | 99.30 (89.29-105.1) | 0.0422^*^ |
| *Cntnap2* KO | Baseline | 88.85 (81.96-92.36) | ̶ |
|  | 75dB prepulse | 100.5 (94.50-105.4) | < 0.0001^****^ |
|  | 80dB prepulse | 92.10 (86.66-100.1) | 0.219^ns^ |
|  | 85dB prepulse | 93.12 (83.82-100.3) | 0.1675^ns^ |
| Median (IQR). Post-hoc: Dunn’s test of multiple comparisons. ^*^*p* < 0.05, ^**^*p* < 0.01, ^***^*p* < 0.001, ^****^*p* < 0.0001, ns: not significant. | | | |

| **Supplementary Table 6 (cont.).** Within genotype comparison of the sound scaling parameter, saturation point. | | | |
| --- | --- | --- | --- |
| **Cohort** | **Comparison** | **Saturation point (dB SPL)** | **Baseline vs Prepulse** |
| *Cntnap2* WT | Baseline | 103.6 (96.59-107.3) | ̶ |
|  | 75dB prepulse | 116.6 (112.1-120.3) | 0.0002^***^ |
|  | 80dB prepulse | 113.4 (103.8-120.2) | 0.0259^*^ |
|  | 85dB prepulse | 116.8 (110.9-123.5) | 0.0008^***^ |
| *Cntnap2* KO | Baseline | 112.5 (100.3-122.2) | ̶ |
|  | 75dB prepulse | 116.2 (110.8-117.9) | ̶ |
|  | 80dB prepulse | 110.7 (105.5-113.0) | ̶ |
|  | 85dB prepulse | 118.1 (109.1-123.6) | ̶ |
| Median (IQR). Post-hoc: Dunn’s test of multiple comparisons. ^*^*p* < 0.05, ^**^*p* < 0.01, ^***^*p* < 0.001, ^****^*p* < 0.0001, ns: not significant. | | | |

**References**

1. Koch M. The neurobiology of startle. Prog Neurobiol. 1999;59(2):107–28.

2. Gomez-Nieto R, Hormigo S, Lopez DE. Prepulse inhibition of the auditory startle reflex assessment as a hallmark of brainstem sensorimotor gating mechanisms. Brain Sci. 2020;10:639.

3. Fulcher N, Azzopardi E, De Oliveira C, Hudson R, Schormans AL, Zaman T, et al. Deciphering midbrain mechanisms underlying prepulse inhibition of startle. Prog Neurobiol. 2020 Feb;185:101734.

4. Weible AP, Yavorska I, Kayal D, Duckler U, Wehr M. A layer 3→5 circuit in auditory cortex that contributes to pre-pulse inhibition of the acoustic startle response. Front Neural Circuits. 2020;14:553208.
